# Return to work after cancer–the impact of working conditions: A Norwegian Register-based Study

**DOI:** 10.1007/s11764-023-01503-0

**Published:** 2023-12-20

**Authors:** Giang Huong Le, Åsmund Hermansen, Espen Dahl

**Affiliations:** Department of Social Work, Child Welfare and Social Policy, OsloMet - Oslo Metropolitan University, Faculty of Social Sciences, Oslo, Norway

**Keywords:** Return to work, Cancer survivors, Working conditions, Job exposure matrix (JEM), Register data

## Abstract

**Purpose:**

The purpose of this study is to compare a cohort of cancer survivors with a cohort of cancer-free employees (1) with respect to employment prospects over a 15-year period and (2) with respect to the differential impact of working conditions on employment over this time period.

**Methods:**

The cancer cohort is retrieved from the Cancer Registry of Norway, while data on the non-cancer cohort are retrieved from register data managed by Statistics Norway. Job exposure matrices were used to remedy the lack of working-conditions information in the register data. We use nearest-neighbor matching to match the non-cancer cohort (the control group) to the cancer-survivor cohort (the treatment group). Cox regression analysis was applied to examine the relationships between working conditions, employment, and cancer. The results are reported separately for mechanical-job exposures and psychosocial exposures, as well as by gender.

**Results:**

Cancer survivors are more likely to experience reduced employment as compared to individuals without a history of cancer. Male cancer survivors in physically demanding occupations have an increased risk of reduced employment after being diagnosed with cancer. This does not apply to female cancer survivors. Regarding the impact of psychosocial exposures on employment, we find no differences over time between cancer survivors and the non-cancer population.

**Conclusions:**

Male cancer survivors in physically demanding occupations have an increased risk of reduced employment after being diagnosed with cancer, whereas this is not the case for female cancer survivors. Psychosocial exposures do not impact the relative risk of reduced employment over time.

**Implications for cancer survivors:**

We suggest that return to work after cancer should be considered a process rather than only the re-entry step of resuming work. Thus, it is important to provide long-term support for cancer survivors. We recommend providing more attention to working conditions, particularly in occupations that involve a high level of mechanical-job exposures.

**Supplementary Information:**

The online version contains supplementary material available at 10.1007/s11764-023-01503-0.

## Introduction

Over the past decades, we have witnessed steady growth in new cancer cases [1]. In Norway, there has been an increase in the number of new cancer cases, with 36,998 new cases being recorded in 2021, representing an increase of over 6,000 cases since 2011 [2]. In Norway, like elsewhere, there has been an increasing trend in long-term cancer survivorship, even for some previously more lethal cancers [3], and in the future, the survival rate for cancer patients is expected to increase further due to early screening and advancements in cancer treatment [4]. The fact that a growing number of long-term cancer survivors are of working age makes the issue of returning to work (RTW) even more pertinent for researchers, as well as policy makers. Specifically, the working conditions that cancer survivors experience may impact their likelihood of resuming or exiting work. Cancer survivors who work under physically and psychosocially demanding conditions may face higher risks of poor health and reduced labor market participation [5–8].

Resumed employment among cancer survivors, as well as other groups who have experienced similar health shocks, are thus high on policymakers’ agenda [9]. In a social democratic welfare state, which relies on a large tax base for its sustainability, high employment rates are an essential prerequisite. For individual workers, employment not only provides earnings and economic wellbeing but also meets psychosocial needs, such as having a time structure, a personal identity, respect, dignity, valued social roles, participation, and a sense of belonging [7, 10]. Given that it is of a certain quality, work may even promote health, in particular mental health, and contribute to recovery from health challenges [11]. Addressing employment after cancer and the impact of working conditions on RTW among cancer survivors, the following research questions guide our study:

o RQ1: Do cancer survivors have an elevated long-term relative risk of reduced employment after being diagnosed with cancer?
o RQ2: Do mechanical and psychosocial working conditions impact the long-term relative risk of reduced employment among cancer survivors?

Research has highlighted determinants of RTW at several levels, such as the individual, the workplace, and the design of welfare and health systems [9]. These factors also consistently include social class, occupation, and education [12, 13]. Studies often show that RTW generally occurs more often among privileged social groups (e.g., the more highly educated [13]). Such socially stratified processes are therefore likely to reinforce social inequalities, as well as health inequalities. However, researchers have rarely investigated the role played by the work environment in RTW processes [14]. This is surprising in light of research knowledge about the relationship between working conditions and health, which may be health promoting or health deteriorating, as well as the well-known association between socioeconomic position, ill health, and exit from work [12, 13, 15]. Because cancer survivors often experience varied long-term side-effects caused by cancer itself or cancer treatment, it is likely that a poor or demanding work environment is a barrier to RTW. People in lower socioeconomic positions (e.g., those in a low social class and those with little education) are particularly vulnerable, partly because they are more often exposed to hazardous working conditions and partly because their opportunities for alternative jobs are limited. Additionally, research shows that less privileged workers more often suffer from co-morbidities [16]. Thus, under such circumstances, a poor work environment may represent an even larger obstacle, and these workers may be facing a situation of “triple jeopardy,” which is likely to significantly reduce the probability of RTW.

In this study, we scrutinize how working conditions are associated with RTW among cancer survivors in Norway. To do so, we analyze longitudinal register data on the entire Norwegian population. The novel contribution of this study is the use of two job exposure matrices (JEMs) that reflect occupation-based mechanical (or physical) and psychosocial job exposures, respectively [17, 18]. As indicated above, this approach allows us to explore whether hazardous working conditions play a role in RTW after cancer diagnosis while also taking into account sociodemographic and socioeconomic factors.

### Conceptual framework

Stergiou-Kita et al. (2014) [19] conducted a meta-synthesis of cancer survivors’ experiences related to obstacles to and opportunities for re-entering work. They reviewed 39 papers on the topic and developed a conceptual framework that is useful for our purpose.

First, the RTW process and the factors framing it are indeed multiple, complex, and found on multiple levels. The factors that are associated with successful RTW can be categorized into three domains: the *personal level*, involving symptoms, work abilities, coping, and motivation; *environmental support*, relating to the workplace, the family, and health care professionals; and the *occupational level*, including the type of work performed, work demands, and job flexibility.

These three conceptual constructs are helpful in identifying crucial factors and establishing links between the various factors and levels. In this way, this conceptual framework will guide our analysis. As important as it is for this review to pinpoint important framing factors, the time dimension itself is missing, even though it is a crucial factor. Young et al. (2005) [20] have proposed that a RTW process includes four phases: 1) Off work, 2) Re-entry, 3) Maintenance, and 4) Advancement. It follows from the long-time perspective of this study (14 years) that the focus is on long-term consequences (i.e., Phase 3, Maintenance, and Phase 4, Advancement).

In their literature review, Stergiou-Kita et al. (2014) [19] also point out certain research gaps. These include, first, the need to address long-term developments, rather than short term transitions pertaining primarily to the re-entry phase. The authors claim that “the vast majority of studies” (p.667) have directed attention to survivors’ early RTW. Thus, there is a need for studies that have a longer timeframe, such as the present study. Second, based on the documented knowledge gaps, they argue that future research should “investigate the influence of job tasks, occupational demands, and work environment, on survivors’ RTW experiences” (p.667), which is exactly what the present study is aiming to accomplish.

Based on this framework, the present study will focus on factors operating on the personal level and, as is especially called for, the occupational level, primarily in terms of working conditions but also in terms of social class. Furthermore, the time horizon is long term and addresses later phases of the RTW process, such as the maintenance and advancement phases.

### Previous research

Current studies on RTW after cancer diagnosis primarily focus on investigating the likelihood of RTW and the associated factors during *the early phases* of the RTW process. The prevailing trend in many countries indicates that cancer survivors, during the re-entry phase, often manage to resume work but are more likely than those who have never had cancer to experience a decline in labor force participation and earnings in the long term [21–28]. Evidence from both the United States and European countries reveals that about 62% of cancer patients RTW within one year of diagnosis and that this figure reaches 89% at 24 months after diagnosis [24]. Notably, Norway has one of the highest rates of RTW among cancer survivors, with 80–90% of survivors returning to employment post-diagnosis [26, 29]. However, the likelihood of RTW differs depending on gender and the specific type of cancer. One study found that, even with the implementation of supportive programs, such as “Rapid-Return to Work,” female cancer survivors still encounter challenges in RTW [30]. Certain types of cancer, such as lung, brain, bone, colorectal, and head-and-neck cancer, as well as treatment with chemotherapy for cancers like leukemia and lymphomas, are associated with higher risks of reduced employment than others [26].

Current research has identified the factors associated with RTW among cancer survivors at various levels. At the *individual level*, factors such as gender, age, marital status, level of education, income, type of cancer, type of treatment, and psychosocial and physical health conditions have been reported to be linked to RTW [24, 31–36]. Furthermore, current studies have documented social stratification in RTW, wherein cancer survivors with lower socioeconomic status face higher risks of unemployment, impaired work ability, reduced working hours, and financial burdens [29, 37–40]. In addition to the individual level, factors related to the *environmental level*, such as workplace accommodation and services received from health or vocational support services, have also been reported to be linked to RTW [41–44]. At the *occupational level*, discrimination at work, low social support, and high work demands are risk factors for not returning to work [7]. Specifically, heavy physical work demands, strenuous work posture and unsupportive work settings have been found to be negatively associated with resuming work after cancer [5, 6, 8, 45].

Although important findings have emerged in previous studies on employment in cancer survivorship, several knowledge gaps exist. First, most studies investigating the effects of occupational factors on RTW have primarily relied on survey or interview data [6, 8, 29, 46–48]. Register data that include entire populations are seldom used, and if they are, information on working conditions has been absent. Second, most cancer survivorship studies have followed up for only 2 or 3 years after diagnosis [34, 44, 49, 50], with only one study, using Norwegian data, that followed up cancer survivors for 9 years [21]. The lack of studies investigating RTW over a long period of time may lead to a limited understanding of RTW across multiple phases, spanning from the early phase (re-entry) to the later phases (retention and maintenance work) after cancer diagnosis and treatment.

Therefore, the primary aim of our study is to compare a cohort of cancer survivors with a cohort of cancer-free employees (1) with respect to employment prospects over a 15-year period and (2) with respect to the differential impact of working conditions on employment over this time period.

We utilize register data to perform a 15-year longitudinal study, considering data from 2006 to 2020, to provide new evidence on the relationship between working conditions and employment after a cancer diagnosis. The study will utilize two job exposure matrices, which will be described in the Methods section, to assess the role of working conditions in the RTW process. Given the evidence regarding social stratification in RTW [39, 41], our study includes position in the socioeconomic hierarchy (i.e., educational level and occupational class), in addition to sociodemographic variables.

### The Norwegian institutional context

An employee with an illness or disease is normally entitled to sickness benefits for 1 year. The first 16 days are paid by the employer, whereas the remaining days are paid by the social security administration (NAV). Sickness benefits are equal to the full salary paid by the employer. There is, however, a cap on the benefit. Salary exceeding six times the base amount of social security (in 2023, 111,477 NOK) is not compensated. After one is certified as sick, work-related activities may be required to receive the benefit. The main rule is that the worker is required to carry out such activities within eight weeks. Within four weeks, the employer and the employee must prepare a follow-up plan.^1^ If the employee is not ready for work after one year, (s)he may be eligible for a work assessment allowance (WAA). To qualify for a WAA, the ability to work must be impaired by at least 50%. A WAA is based on the pensionable income earned the year before the capacity to work was reduced. A WAA can be received for up to 3 years, and the person is entitled to 66% of the earned income, with a cap of six times the base amount. To have the right to a WAA benefit, the recipient must comply with “the duty to act.” This involves developing an activity plan describing the steps needed to RTW.^2^ If RTW fails after a certain period of time, the person may be eligible for a permanent disability benefit.

### Data, study population, and methods

#### Data

In this study, we used the Cancer Registry of Norway (Kreftregisteret) as a source for our cancer cohort. The Cancer Registry of Norway is a comprehensive database that is composed of various sources of information, including clinical notifications, pathology reports, and national registries such as the Norwegian Patient Registry and the Cause of Death Registry. These data are linked through the personal identification number system [2]. The cancer-free population is retrieved from register data managed by Statistics Norway (Statistisk sentralbyrå). The register data from Statistics Norway also include information on demographics, education, occupation (the basis for social class), and income for the entire population used in our study.

#### Study population: identifying cancer survivors and non-cancer-survivors

A cancer-survivors cohort was established based on the following conditions: (1) diagnosed with cancer for the first time between January and December 2007; (2) survived for the long term, meaning at least 13 years after their cancer diagnosis; (3) aged between 30 and 50 at the time of diagnosis (born between 1977 and 1957)^3^; (4) employed for a full 12 months prior to the year of diagnosis (year 2006) and did not receive disability benefits or a WAA during that time; and (5) had valid occupational codes. Given these criteria, our cancer-survivor cohort consisted of 899 individuals, out of the 23,465 new cancer cases in 2007.

A control group of cancer-free individuals was also established. The control group met the same criteria as the cancer group regarding age, employment, valid occupational codes, and benefits received in 2006. After merging the variables of interest in the data, we combined data from both the cancer and the non-cancer cohorts, resulting in a total sample size of 430,701 individuals.

#### Independent variables

##### Cancer status

Was created as a dichotomy, with a value of 0 for individuals in the non-cancer cohort and 1 for those included in the cancer cohort.

##### Socioeconomic status

Was represented by two variables: education and occupational class.

The education variable measures the highest level of education achieved by the individual, which is (1) Secondary education and lower; (2) High school education; (3) University and college with a 4-year degree; or (4) University and college for more than 4 years, including a master’s degree or doctoral degree.

The occupational class was recoded based on the occupational codes, following the Norwegian standard for occupation classification (STYRK-08), which is based on the International Standard Classification of Occupations (ISCO-08). First, eight groups of occupational classes were created, namely the higher salariat, the lower salariat, higher-grade white-collar workers, petit bourgeoisie or independent, higher-grade blue-collar workers, lower-grade white-collar workers, skilled workers, and semi- and unskilled workers, using the European Socio-economic Classification [51]. After that, three major occupational classes were established as dummy variables, namely (1) upper-non-manual, including higher salariat and higher-grade white-collar workers; (2) non-manual, including lower salariat, petit bourgeoisie or independent, lower-grade white-collar, higher-grade blue-collar, and skilled workers; and (3) manual, including semi- and unskilled and lower-grade blue-collar workers.

##### Working conditions

were measured by using the Occupational Mechanical Job Exposure Index and Occupational Job Strain Index, which were constructed and validated for use in register-based research [17, 18]. The two job exposure indices were based on five Norwegian nationwide surveys of living conditions concerning work environment in 2006, 2009, 2013, 2016, and 2019. The mechanical index includes eight mechanical-exposure items.^4^ The strain index is a combination of the psychological demand index (job demand), including four items,^5^ and decision-latitude index (job control), including six items.^6^ All the exposure variables were constructed as the proportion of individuals within occupational groups that are exposed to the specific exposures. The mechanical index measures the mean proportion of mechanical exposures within each occupation and theoretically ranges from 0 to 100%. The value 0 implies that no one has reported being exposed as part of a given occupation, whereas a value of 100 implies that everyone with a given occupational code has reported being exposed to all eight mechanical exposures. The strain index also theoretically ranges from 0 to 100 percent, where higher values on the index represent higher degrees of demand and lower degrees of control. For a more detailed description of the mechanical index, see Hermansen and Dahl (2022) [17]. See Le, Hermansen, and Dahl (2023) [18] for a description of the job strain index.

### Outcome variable

We measured employment using annual income from work between 2006 to 2020 and 3.5 times the basic amount (known as *grunnbeløpet (G)* in Norwegian) as a cut-off. The income variable represents annual pre-taxed income from work and is inflation-adjusted over the study period (2006–2020). The income considers the income earned by individuals regardless of whether they are engaged in full-time or part-time employment. The basic amount (G) is adjusted annually by the Government^7^ and forms the basis for calculating many of the NAV’s benefits, including pensions and disability benefits. The basic amount in 2020 was 101,351 NOK, corresponding to an annual income from work of 354,729 NOK or more when using cut-off of 3.5 basic amounts. An income of 3.5 basic amounts correspond to the yearly income from work for a full-time employee in the lowest income bracket.

### Propensity score matching

To reduce bias in the comparison of groups, propensity score matching (PSM) was applied to the entire sample using nearest-neighbor matching. One case from the cancer group could be matched to multiple cases in the control group (1: n), with replacement, and a caliper width of 0.2 was used [52]. A variety of cofounding variables were considered in the matching process, including gender; country of origin; age; marital status; total income from work in 2006, including sick pay and maternity benefits; and total benefit received in 2006, including the parental allowance, the child benefit, the housing benefit, social assistance, the basic and auxiliary allowance, and cash support.

When faced with the choice between 1:1 (one–one) matching or 1:n (one-many) matching, we opted for the latter method. Although 1:n matching may lead to less common support between the two samples, as shown in Figure 3, in the Appendix, it helps to preserve a larger total sample size, which may enhance the external validity of our study’s results. Furthermore, using a 1:n matching approach in cohort studies is recommended [53]. The choice of multiple cases 'n' in 1:n matching was made to enhance the precision of our estimates and increase the robustness of our findings, especially when we have access to a large pool of potential controls in our dataset. Given the research goals, the purpose of this study is to compare a cohort of cancer survivors with a cohort of cancer-free employees based on register data spanning over a 15-year period. This approach is expected to yield meaningful results, particularly due to the substantial number of control cases available.

The results of a t-test (Table 6, Appendix) indicate that there are no significant differences between the treatment and control groups after matching, as all p-values are greater than 0.05. Although the quality of matched pairs, as shown in common support result, is not ideally overlapping, the results comparing the means between two samples (Table 6 and Figure 2, Appendix) demonstrate that these two samples do not differ significantly regarding confounding factors. After matching, 167,921 individuals were omitted from the study. The final sample consisted of 262,780 individuals.

### Analytical strategy

We first performed a descriptive analysis of the demographics of both the cancer cohort and non-cancer cohort, including information on gender, age, country of origin, educational level, occupational class, and cancer site (for the cancer cohort). Secondly, we evaluated the proportional hazard (PH) assumption by using log(-log) plots, which is commonly used to investigate the potential violation of the PH assumption [54]. Then, we used Cox regression to examine the relationships between cancer status, working condition and work outcome in gender-specific runs. Given the potential variation in cancer cohort entry risk by social class, including education and occupation, we included education and occupational class as control variables in our model. Furthermore, to examine whether working conditions operated as moderators between cancer status and employment (RQ 2), we added interaction terms between the working-conditions variables and cancer status. The results are reported separately based on the types of working conditions, which encompass the mechanical job exposure index and the strain index. In light of previous evidence suggesting gender differences in RTW [40, 55], these analyses are performed separately for men and women.

## Results

A total of 262,780 individuals were included in the study, consisting of 899 cancer survivors and 261,881 individuals without cancer. Table 1 provides the background characteristics for the study population.

**Table 1:** Descriptive statistics for the sample (*N* = 262,780)

	Non-Cancer		Cancer		Total	
	N	*%*	N	*%*	N	*%*
Gender						
Male	110,645	*42.25*	336	*37.37*	110,981	*42.23*
Female	151,236	*57.75*	563	*62.63*	151,799	*57.77*
Age groups						
30–40	107,848	*41.18*	302	*33.59*	108,150	*41.16*
41–50	154,033	*58.82*	597	*66.41*	154,630	*58.84*
Country background						
Norway	242,495	*92.60*	836	*92.99*	243,331	*92.60*
EU/EEA	8,571	*3.25*	30	*3.34*	8,547	*3.25*
Others	10,896	*4.15*	33	*3.67*	10,902	*4.15*
Education (highest level)						
Secondary education and lower	51,059	*19.50*	182	*20.24*	51,241	*19.50*
High school education	125,202	*47.81*	370	*41.16*	125,572	*47.79*
University and college (4 years)	71,415	*27.27*	278	*30.92*	71,693	*27.28*
University and college (> 4 years)	14,205	*5.42*	69	*7.68*	14,274	*5.43*
Occupational class						
Upper non-manual	76,926	*29.37*	283	*31.48*	77,209	*29.38*
Non-manual	148,021	*56.52*	514	*57.17*	148,535	*56.52*
Manual	36,934	*14.10*	102	*11.35*	37,036	*14.09*
Cancer sites						
Digestive organs	-	*-*	64	*7.12*	-	*-*
Respiratory and intrathoracic organs	-	*-*	18	*2.00*	-	*-*
Skin, including melanoma	-	*-*	89	*9.90*	-	*-*
Breast	-	*-*	243	*27.03*	-	*-*
Female genitalia	-	*-*	77	*8.57*	-	*-*
Male genitalia	-	*-*	100	*11.12*	-	*-*
Urinary tract	-	*-*	22	*2.45*	-	*-*
Eye, brain, and other part of central nervous system	-	*-*	99	*11.01*	-	*-*
Thyroid and other endocardial	-	*-*	63	*7.01*	-	*-*
Others	-	*-*	124	*13.79*	-	*-*

Among the cancer patients diagnosed for the first time in 2007, the majority were females aged 41 to 50, originating from Norway, with high school as their highest level of education, and the majority are in the non-manual class. The most prevalent types of cancer were breast (27.03%); genitalia (19.69% for both male and female); eye, brain, and other parts of central nervous system (11.01%); and skin (9.90%). As we are examining cancer as a whole, further analysis will not take specific cancer sites into account as a variable of interest.

**Fig. 1:**
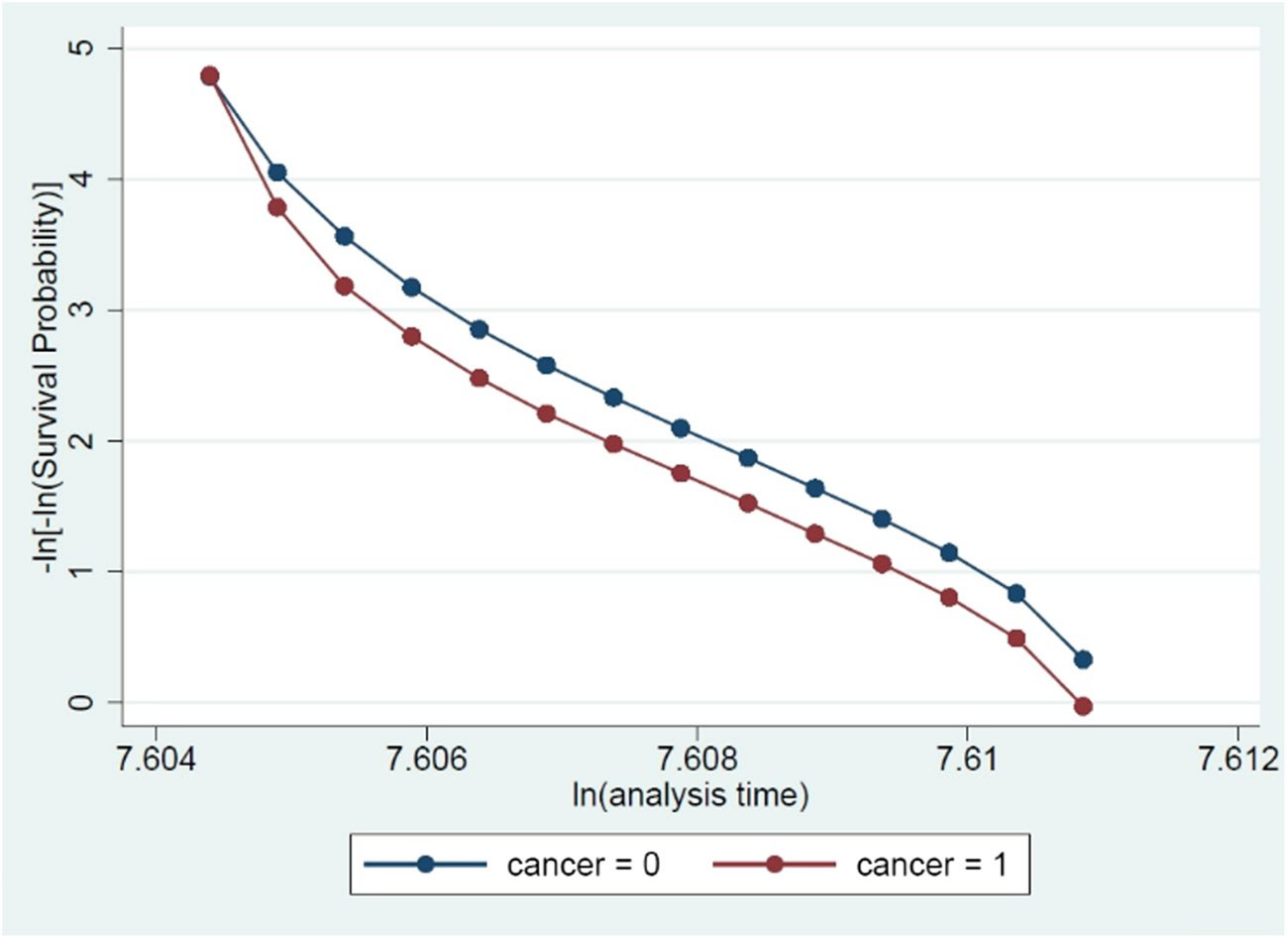
Testing PH assumption using log–log plots

Figure 1 presents the log–log plots used to assess the PH assumption by comparing the estimated -In(-In) survival curves. The curves remain parallel, indicating that the PH assumption is unviolated. The graph shows that individuals with cancer have a higher relative risk of reduced employment (income from work ≤ 3.5 G) as compared with those without cancer; however, the variances between the two groups are relatively small.

Tables 2, 3, 4 and 5 presents the findings derived from the Cox regressions examining the impact of working conditions, which are reported using the Mechanical Job Exposure Index (Tables 2 and 3) and the Job Strain Index (Tables 4 and 5), on RTW after cancer diagnosis for both genders. In these tables, Model 1 only includes cancer and socioeconomic status variables; in Model 2, working-condition variables (mechanical index/strain index) are added, and Model 3 introduces the interaction terms between the mechanical index and cancer and the strain index and cancer to the variables in Model 2.

**Table 2:** Results of Cox regression analysis with employment (> 3.5 basic amounts in income from work) as outcome variable^a^; models included the predictor variables cancer^b^, education level^c^, occupational class^d^, mechanical job exposure (MJE) and the interaction term between MJE and cancer. Results reported for men

Variables	Model 1	Model 2	Model 3
	Hazard ratio (95%CI)	Hazard ratio (95%CI)	Hazard ratio (95%CI)
Cancer	1.27*** (1.17–1.38)	1.28*** (1.19–1.39)	0.98 (0.84–1.15)
Education: High school	0.61*** (0.60–0.61)	0.61*** (0.60–0.61)	0.61*** (0.60–0.61)
Education: University and college (4 years)	0.46*** (0.46–0.47)	0.49*** (0.48–0.50)	0.49*** (0.48–0.50)
Education: University and college (> 4 years)	0.28*** (0.27–0.29)	0.30*** (0.29–0.31)	0.30*** (0.287–0.31)
Class: Manual	1.65*** (1.63–1.68)	1.51*** (1.48–1.53)	1.51*** (1.48–1.53)
Class: Non-manual	1.27*** (1.26–1.29)	1.13*** (1.11–1.15)	1.13*** (1.11–1.15)
Mechanical job exposure index		1.89*** (1.83–1.97)	1.89*** (1.82–1.96)
MJE*cancer			2.95*** (1.75–4.95)

^a^Outcome variable: 0 = income from work > 3.5G, 1 = income from work ≤ 3.5G

^b^Cancer: 0 = non-cancer, 1 = cancer

^c^Education: secondary and lower is reference group

^d^Occupational class: upper non-manual is reference group

^*^ p: probability value (*** *p* < 0.001, ** *p* < 0.01, * *p* < 0.05); 95%CI: 95% confidence interval

**Table 3:** Results for Cox regression analysis with employment (> 3.5 basic amounts in income from work) as outcome variable^a^; models included the predictor variables cancer^b^, education level^c^, occupational class^d^, mechanical job exposure (MJE), and the interaction term between MJE and cancer. Results reported for women

Variables	Model 1	Model 2	Model 3
	Hazard ratio (95%CI)	Hazard ratio (95%CI)	Hazard ratio (95%CI)
Cancer	1.42*** (1.37–1.48)	1.44*** (1.38–1.49)	1.43*** (1.31–1.55)
Education: High school	0.71*** (0.70–0.71)	0.71*** (0.70–0.71)	0.71*** (0.70–0.71)
Education: University and college (4 years)	0.36*** (0.36–0.37)	0.36*** (0.36–0.37)	0.36*** (0.36–0.37)
Education: University and college (> 4 years)	0.22*** (0.22–0.23)	0.25*** (0.24–0.25)	0.25** (0.24–0.25)
Class: Manual	1.82*** (1.80–1.84)	1.38*** (1.36–1.40)	1.38*** (1.36–1.40)
Class: Non-manual	1.54*** (1.53–1.55)	1.24*** (1.23–1.25)	1.24*** (1.23–1.25)
Mechanical Job Exposure Index		3.79*** (3.67–3.91)	3.79*** (3.67–3.91)
MJE*cancer			1.03 (0.74–1.44)
^a^Outcome variable: 0 = income from work > 3.5G, 1 = income from work ≤ 3.5G ^b^Cancer: 0 = non-cancer, 1 = cancer ^c^Education: secondary and lower is reference group ^d^Occupational class: upper non-manual is reference group *p: probability value (*** *p* < 0.001, ** *p* < 0.01, * *p* < 0.05); 95%CI: 95% confidence interval

**Table 4:** Results for Cox regression analysis with employment (> 3.5 basic amounts in income from work) as outcome variable^a^; models included the predictor variables cancer^b^, education level^c^, occupational class^d^, job strain index (JSI), and the interaction term between JSI and cancer. Results reported for men

Variables	Model 1	Model 2	Model 3
	Hazard ratio (95%CI)	Hazard ratio (95%CI)	Hazard ratio (95%CI)
Cancer	1.27*** (1.17 – 1.38)	1.27*** (1.17 – 1.38)	1.12 (0.75 – 1.67)
Education: High school	0.61*** (0.60—0.61)	0.61*** (0.61—0.62)	0.61*** (0.61—0.62)
Education: University and college (4 years)	0.46*** (0.46—0.47)	0.46*** (0.45—0.46)	0.46*** (0.45—0.46)
Education: University and college (> 4 years)	0.28*** (0.27—0.29)	0.28*** (0.27—0.29)	0.28*** (0.27—0.29)
Class: Manual	1.65*** (1.63 – 1.68)	1.41*** (1.38 – 1.43)	1.41*** (1.20 – 1.24)
Class: Non-manual	1.27*** (1.26 – 1.29)	1.22*** (1.20 – 1.24)	1.22*** (1.21 – 1.24)
Job Strain Index		6.21*** (5.67 – 6.80)	6.20*** (5.66 – 6.79)
JSI*cancer			1.53 (0.43 – 5.40)

^a^Outcome variable: 0 = income from work > 3.5G, 1 = income from work ≤ 3.5G

^b^Cancer: 0 = non-cancer, 1 = cancer

^c^Education: secondary and lower is reference group

^d^Occupational class: upper non-manual is reference group

* p: probability value (*** *p* < 0.001, ** *p* < 0.01, * *p* < 0.05); 95%CI: 95% confidence interval

**Table 5:** Results for Cox regression analysis with employment (> 3.5 basic amounts in income from work) as outcome variable^a^; models included the predictor variables cancer^b^, education level^c^, occupational class^d^, job strain index (JSI), and the interaction term between JSI and cancer. Results reported for women

Variables	Model 1	Model 2	Model 3
	Hazard ratio (95%CI)	Hazard ratio (95%CI)	Hazard ratio (95%CI)
Cancer	1.42*** (1.37–1.48)	1.43*** (1.37–1.48)	1.47** (1.12–1.92)
Education: High school	0.71*** (0.70–0.71)	0.71*** (0.70–0.71)	0.71*** (0.70–0.71)
Education: University and college (4 years)	0.36*** (0.36–0.37)	0.36*** (0.357–0.363)	0.36*** (0.357–0.363)
Education: University and college (> 4 years)	0.22*** (0.22–0.23)	0.22*** (0.22–0.23)	0.22*** (0.22–0.23)
Class: Manual	1.82*** (1.80–1.84)	1.76*** (1.74–1.78)	1.76*** (1.74–1.78)
Class: Non-manual	1.54*** (1.53–1.55)	1.51*** (1.50–1.52)	1.51*** (1.50–1.52)
Job Strain Index		1.82*** (1.71–1.93)	1.82*** (1.71–1.93)
JSI*cancer			0.92 (0.42–2.00)

^a^Outcome variable: 0 = income from work > 3.5G, 1 = income from work ≤ 3.5G

^b^Cancer: 0 = non-cancer, 1 = cancer

^c^Education: secondary and lower is reference group

^d^Occupational class: upper non-manual is reference group

* p: probability value (*** *p* < 0.001, ** *p* < 0.01, * *p* < 0.05); 95%CI: 95% confidence interval

The Cox regression analysis results presented in Tables 2 and 3 reveal that cancer survivors have a higher relative risk of reduced employment over time as compared with those without cancer. This is true for both men and women (HR > 1, *p* < 0.001 for both genders). Male cancer survivors exhibit a 27% greater hazard rate than their male counterparts without a cancer diagnosis, while female cancer survivors have a 42% greater hazard rate than female non-cancer-survivors. Moreover, individuals with a lower education level (i.e., high school) and those belonging to the manual occupational class are more likely to experience a higher relative risk of reduced employment over time. The results from Model 2 in Tables 2 and 3 indicate that males who work in occupations with high mechanical job exposure have a 1.89-fold greater relative risk of reduced employment (HR = 1.89, *p* < 0.001), while females working in such jobs have a 3.79-fold higher relative risk (HR = 3.79, *p* < 0.001). Furthermore, Model 3 reveals a significant interaction effect between the Mechanical Job Exposure Index and cancer for men (*p* < 0.001; Table 2), but this is not true for women (*p* > 0.05; Table 3), suggesting that the impact of cancer on employment varies depending on the level of mechanical job exposures among male workers.

The Cox regression analysis results presented in Tables 4 and 5 indicate that higher levels of job strain increase the relative risk of reduced employment for both men and women. However, the results of Model 3 in Table 4 and Table 5 reveal that job strain does not appear to moderate the relationship between cancer and employment.

In summary, our study shows that, among the four interaction terms between working conditions and cancer, for men and women, only mechanical job exposures significantly impacts the relative risk of reduced employment for male cancer survivors. Due to the known association between working conditions and education and social class, we carried out an additional analysis without these two socioeconomic variables. This analysis shows that the original results shown in Table 2, 3, 4 and 5 do not change,^8^ lending credibility to the finding that only mechanical job exposures among men are related to return to work.

## Discussion

The results of this study indicate that cancer survivors are more likely to experience a higher relative risk of reduced employment as compared to individuals without a history of cancer, which is in line with most previous studies [25–27, 56–58]. These results are also consistent for lower cut-off values of the income-from-work variable used a measure of employment. The results for a cut-off value > 1 basic amount and > 0.5 basic amounts are shown in Appendix Tables 7–14. Furthermore, our findings support the notion that working conditions play a vital role in RTW after cancer [48]. Male cancer survivors in physically demanding jobs have significantly a higher relative risk of reduced employment as compared with their cancer-free peers.

Our results reveal a gender difference among cancer survivors in terms of mechanical job exposure, but this is not the case for job strain. This result is intuitive in the sense that physical demanding jobs are commonly held by men due to horizontal segregation in the labor market, which places men in the occupations that are more likely to be exposed to physical hazards, rather than women [59]. Furthermore, in cases in which men and women have equivalent job positions, men typically undertake more physically strenuous tasks, leading to greater physical demands for men in comparison to women [60]. Additionally, cancer treatment often results in long-term fatigue, posing a challenge for individuals attempting to manage in such jobs. Surprisingly, our results demonstrate that psychosocial work hazards do not play a role in RTW after cancer for any gender, as compared with cancer-free employees. These findings are consistent with a prior study conducted in Norway [61] that suggested no significant association between cancer survivorship status and job strain, as well as no significant differences between female and male survivors in terms of job strain scores. Therefore, our study suggests that physical work demands may have a stronger impact on RTW post-diagnosis as compared to psychosocial work demands. A plausible explanation for this phenomenon is that cancer survivors may effectively cope with the psychosocial aspects of work by adjusting in terms of work arrangements, as well as receiving support from colleagues upon their return, but managing intense physical tasks may prove more challenging for those who work in physically demanding occupations. Our study also highlights the fact that lower-class status and short education, for both genders, imply a higher risk of non-employment regardless of cancer and working conditions, underscoring the importance of socioeconomic position regarding labor market activity in general [13].

Our findings reinforce the theoretical framework proposed by Stergiou-Kita et al. (2014) [19], which emphasizes the role of personal and occupational level, including social class and working conditions, in the later phases of RTW. Although cancer survivors likely resume work in the early phase of RTW after treatment, they often experience a reduced physical work capacity due to pain, fatigue, or cognitive dysfunction [19]. It is therefore important to provide long-term support to cancer survivors not only during the re-entry phase but also throughout the maintenance and advancement phases to help them attain living conditions comparable to those during their pre-diagnosis state and those of their counterparts without a history of cancer [62]. Moreover, we should consider whether individuals, regardless of their specific work conditions, may opt to reduce their labor market participation as a purely voluntary choice. This raises important questions about the interplay between health, work, and individual choices that warrant further investigation in future research.

This study emphasizes the importance of considering working conditions, particularly physically demanding work, when developing interventions to support individuals RTW after cancer. We suggest that RTW after cancer should be considered a process rather than merely the re-entry step of resuming work [20]. We also recommend that the RTW process be individually tailor made. For example, individual physical exercise plans before and during process of RTW may be important in improving fitness and energy levels, which can reduce fatigue and help those in physically demanding job maintain their normal employment, as well as improving their quality of life after treatment [63]. The RTW process should consider not only an individual’s health condition and motivation to work but also the specific working conditions they may face upon their RTW. It is important to ensure that cancer survivors have the necessary accommodations and resources to facilitate their RTW while dealing with their impaired health. Thus, the emphasis, during support, should be placed on flexible work arrangement and work tasks that are less physically demanding.

### Strengths and weaknesses of the study

To our knowledge, this is the first longitudinal study conducted in Norway that uses register data spanning a 15-year period to investigate the impact of working conditions on RTW following a cancer diagnosis. Income from work, which was obtained from reliable register data, was utilized to measure employment. In general, Norwegian register data are reliable and accurate and do not suffer from problems related to attrition, which is a challenge in most survey data. However, our study has certain limitations. *First*, we utilized data from the Norwegian population, which limits its comparability to other countries with a similar context, such as the countries with the same welfare system. The study only included individuals aged between 30 and 50 years old, and therefore, the results cannot be generalized to the entire population. The truncated age span also means that the number of cancer survivors is limited (< 1,000), implying that, in the gender-specific analysis, the statistical power to detect significant interactions terms is restricted. Thus, the interactions terms should be interpreted with some caution. Because we did not include people over 50 years of age, further studies may focus on this group. *Second*, the propensity score-matching method is employed to reduce potential cofounder bias [64, 65]. We matched a treatment group with a similar control group based on various cofounders, including gender, country of origin, age, marital status, previous earnings, and benefits received in 2006. However, it is important to note that the effectiveness of propensity score matching depends on the accuracy of a PSM model, which may be affected by unobserved cofounding variables that were not included in our study. To preserve the large amount of data and enhance the external validity of our study, we applied a 1:n nearest-neighbor matching method. Although this method may help to prevent the removal of a substantial amount of data, it may also reduce the internal validity of the study. *Third*, the study applied job exposure matrices, which allow us to investigate the role of working conditions on RTW after cancer based on register data. The two matrices have been validated and found to be reproducible and efficient tools for use in evaluating the mechanical and psychosocial work environment in Norwegian data [17, 18]. Nonetheless, it is important to note that JEMs cannot be seen as a gold standard measure for job exposure [66, 67], as the method entails the risk of errors in JEM assignments [68] and the risk of differential misclassification [66]. *Fourth*, while our study focuses on the relationship between cancer diagnosis, employment outcomes, and working conditions among long-term survivors, it is important to recognize certain limitations. We did not differentiate between individuals with one or multiple cancer diagnoses, limiting our ability to draw conclusions regarding the specific effects of additional cancer diagnoses on work outcomes. Additionally, we did not explore variations related to specific cancer sites and stages. Future studies may benefit from a more detailed analysis of these factors to provide a comprehensive understanding of how they influence employment outcomes among cancer survivors.

## Supplementary Information

Below is the link to the electronic supplementary material.

Supplementary file1 (DOCX 397 KB)

## Data Availability

The data that support the findings of this study are available from Statistics Norway, but restrictions apply to the availability of these data, which were used under license for the current study, and so are not publicly available. Data are however available from the authors upon reasonable request and with permission of the Norwegian Data Protection Official for the Research (NSD) and the Norwegian Data Protection Authority (Datatilsynet).

## Footnotes

1 Sickness benefit for employees: https://www.nav.no/en/home/benefits-and-services/Sickness-benefit-for-employees

2 Work assessment allowance (AAP): https://www.nav.no/aap/en

3 The age range between 30 and 50 was chosen because it represents individuals who of working age and cancer incidence is known to be particularly prevalent among individuals around the age of 40 and above. Also, individuals under the age of 30 may still be in the process of completing their education [21]. Furthermore, we follow cancer survivors over 15 years, from the year prior to diagnosis (2006) to 2020. If we expanded the age limit to over 50, this would mean that the individuals in our sample were close to or over the retirement age, and this could be a reason that they worked less, beyond and cancer and working conditions.

4 Heavy lifting, working with the hands above shoulder height, performing heavy physical labor, neck flexion, squatting/kneeling, forward bending, awkward lifting, and standing/walking.

5 Quantitative demands, conflicting ways of doing things, insufficient resources, and contradictory requests.

6 Decide how to go about the work, decide the pace of work, make important decisions, use skills, develop skills, and monotonous work.

7 For more information on the basic amount: https://www.nav.no/grunnbelopet

8 The results can be provided by request to the corresponding author.
