# Supplementary material for: Return to work after cancer–the impact of working conditions: A Norwegian Register-based Study
Source: J Cancer Surviv. 2023 Dec 20;19(3):766–78. doi: 10.1007/s11764-023-01503-0 (PMC12081524; doi:10.1007/s11764-023-01503-0)
Supplement: Supplementary file 1 — Supplementary file1 (DOCX 397 KB) [file 11764_2023_1503_MOESM1_ESM.docx]

**Article title:** Return to work after cancer–the impact of working conditions: A Norwegian Register-based Study.

**Submit to journal:** Journal of Cancer Survivorship

## **Information of authors**

Giang Huong Le, corresponding author

Title: PhD candidate

Affiliation: OsloMet - Oslo Metropolitan University, Faculty of Social Sciences, Department of Social Work, Child Welfare and Social Policy

Email: [gianghuo@oslomet.no](mailto:gianghuo@oslomet.no)

Address: Postboks 4, St. Olavs plass, 0130 Oslo

ORCID iD: 0000-0003-3261-5088

Åsmund Hermansen

Title: Professor

Affiliation: OsloMet - Oslo Metropolitan University, Faculty of Social Sciences, Department of Social Work, Child Welfare and Social Policy

Email: [asmuhe@oslomet.no](mailto:asmuhe@oslomet.no)

ORCID iD: 0000-0002-0826-0689

Espen Dahl

Title: Professor

Affiliation: OsloMet - Oslo Metropolitan University, Faculty of Social Sciences, Department of Social Work, Child Welfare and Social Policy

Email: [espendah@oslomet.no](mailto:espendah@oslomet.no)

ORCID iD: 0000-0002-3744-2123

# APPENDIX

| Table 6: Result of propensity score matching, comparing unmatched sample and matched sample | | | | | | | |
| --- | --- | --- | --- | --- | --- | --- | --- |
| Variables | Sample | Mean | | %bias | %reduct bias | t-test | |
|  |  | Treated | Control |  |  | t | p |
| Gender | Unmatched | 1.63 | 1.54 | 17.0 |  | 5.02 | 0.00 |
|  | Matched | 1.63 | 1.63 | -1.2 | 92.8 | -0.26 | 0.79 |
| Country origin | Unmatched | .107 | .14 | -7.5 |  | -2.09 | 0.04 |
|  | Matched | .107 | .105 | 0.4 | 94.2 | 0.10 | 0.92 |
| Age | Unmatched | 1.66 | 1.48 | 37.6 |  | 10.96 | 0.00 |
|  | Matched | 1.66 | 1.66 | -0.1 | 99.8 | -0.01 | 0.99 |
| Marital status | Unmatched | 1.83 | 1.75 | 12.6 |  | 3.83 | 0.00 |
|  | Matched | 1.83 | 1.85 | -2.5 | 80.0 | -0.52 | 0.60 |
| Work income (2006) | Unmatched | 3.8e+05 | 3.6e+05 | 10.5 |  | 3.33 | 0.001 |
|  | Matched | 3.8e+05 | 3.7e+05 | 6.3 | 40.3 | 1.29 | 0.19 |
| Benefit received (2006) | Unmatched | 13340 | 15395 | -7.6 |  | -2.05 | 0.04 |
|  | Matched | 13340 | 13868 | -1.9 | 74.3 | -0.46 | 0.64 |


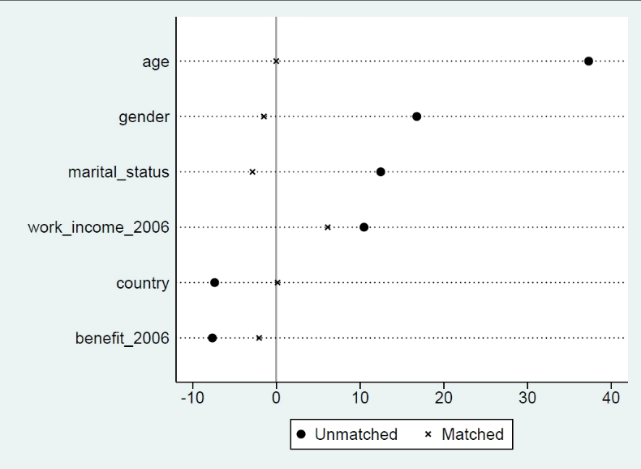


Figure 2: A comparison of unmatched and matched sample.


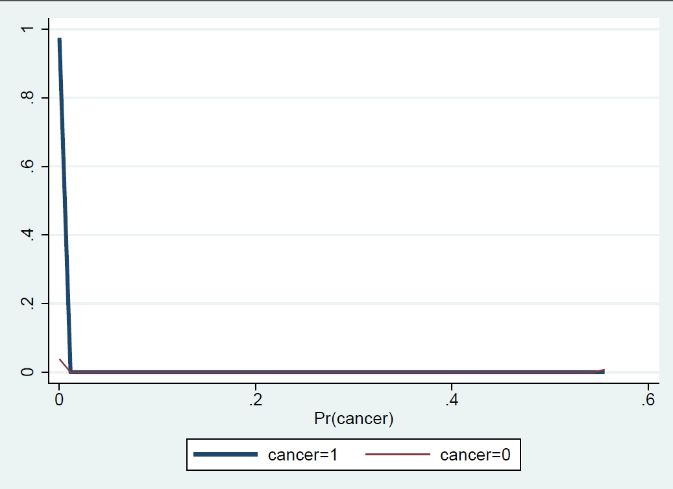

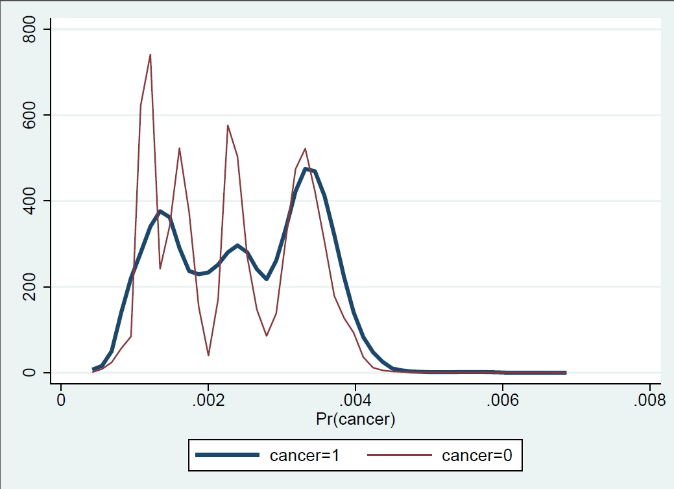


Figure 3: Common support before (left) and after (right) applying nearest neighbor matching.

| Table 7: Results for Cox regression analysis with work (>1.0 basic amounts in income from work) as outcome variable^a^, models included predictor variables cancer^b^, education level^c^, occupational class^d^, mechanical job exposure (MJE) and interaction term between MJE and cancer. Results reported for men | | | |
| --- | --- | --- | --- |
| Variables | Model 1 | Model 2 | Model 3 |
|  | Hazard ratio  (95%CI) | Hazard ratio  (95%CI) | Hazard ratio  (95%CI) |
| Cancer | 1.37***  (1.23 – 1.52) | 1.39***  (1.25 – 1.54) | 1.06  (.87 – 1.32) |
| Education: High school | .56***  (.55 - .57) | .55***  (.55 - .56) | .56***  (.55 - .56) |
| Education: University and colleges (4 years) | .42***  (.41- .43) | .44***  (.43 - .45) | .44***  (.43 - .46) |
| Education: University and colleges (>4 years) | .27***  (.26 - .28) | .29***  (.28 - .30) | .29***  (.28 – 30) |
| Class: Manual | 1.71***  (1.67 – 1.75) | 1.54***  (1.50 – 1.58) | 1.54***  (1.50 – 1.58) |
| Class: Non-manual | 1.27***  (1.24 – 1.29) | 1.11***  (1.09 – 1.14) | 1.11***  (1.09 – 1.14) |
| Mechanical job exposure index |  | 2.03***  (1.93 – 2.14) | 2.03***  (1.93 – 2.13) |
| MJE*cancer |  |  | 2.86***  (1.44 – 5.68) |
| ^a^Outcome variable: 0= Not having risk of low employment (work income >1G), 1= Having risk of low employment (work income ≤1G)  ^b^Cancer: 0 = non-cancer, 1 = cancer  ^c^Education: Secondary and lower is reference group.  ^d^Occupational class: Upper non-manual is reference group.  ^*^ p: probability value (*** p< 0.001, ** p<0.01, * p<0.05); 95%CI: 95% confidence interval | | | |

| Table 8: Results for Cox regression analysis with work (>1.0 basic amounts in income from work) as outcome variable^a^, models included predictor variables cancer^b^, education level^c^, occupational class^d^, mechanical job exposure (MJE) and interaction term between MJE and cancer. Results reported for women | | | |
| --- | --- | --- | --- |
| Variables | Model 1 | Model 2 | Model 3 |
|  | Hazard ratio  (95%CI) | Hazard ratio  (95%CI) | Hazard ratio  (95%CI) |
| Cancer | 1.62***  (1.51 – 1.73) | 1.63***  (1.52 – 1.74) | 1.53***  (1.33 – 1.76) |
| Education: High school | .64***  (.63 - .65) | .64***  (.63 – 65) | .64***  (.63 - .65) |
| Education: University and colleges (4 years) | .38***  (.38 - .39) | .38***  (.38 - .39) | .38***  (.38 - .39) |
| Education: University and colleges (>4 years) | .26***  (.25 - .27) | .28***  (.27 - .29) | .28***  (.27 - .29) |
| Class: Manual | 1.84***  (1.81 – 1.88) | 1.60***  (1.56 – 1.63) | 1.60***  (1.56 – 1.63) |
| Class: Non-manual | 1.28***  (1.27 – 1.3) | 1.15***  (1.13 – 1.17) | 1.15***  (1.13 – 1.17) |
| Mechanical job exposure index |  | 2.00***  (1.88 – 2.12) | 1.99***  (1.88 – 2.11) |
| MJE*cancer |  |  | 1.33  (.76 – 2.32) |
| ^a^Outcome variable: 0= Not having risk of low employment (work income >1G), 1= Having risk of low employment (work income ≤1G)  ^b^Cancer: 0 = non-cancer, 1 = cancer  ^c^Education: Secondary and lower is reference group.  ^d^Occupational class: Upper non-manual is reference group.  * p: probability value (*** p< 0.001, ** p<0.01, * p<0.05); 95%CI: 95% confidence interval | | | |

| Table 9: Results for Cox regression analysis with work (>1.0 basic amounts in income from work) as outcome variable^a^, models included predictor variables cancer^b^, education level^c^, occupational class^d^, job strain index (JSI) and interaction term between JSI and cancer. Results reported for men | | | |
| --- | --- | --- | --- |
| Variables | Model 1 | Model 2 | Model 3 |
|  | Hazard ratio  (95%CI) | Hazard ratio  (95%CI) | Hazard ratio  (95%CI) |
| Cancer | 1.37***  (1.23 – 1.52) | 1.37***  (1.24 – 1.53) | .95  (.56 – 1.62) |
| Education: High school | .56***  (.55 - .57) | .56***  (.55 - .57) | .56***  (.55 - .57) |
| Education: University and colleges (4 years) | .42***  (.41- .43) | .41***  (.40 - .42) | .41***  (.40 - .42) |
| Education: University and colleges (>4 years) | .27***  (.26 - .28) | .27***  (.26 - .28) | .27***  (.26 - .28) |
| Class: Manual | 1.71***  (1.67 – 1.75) | 1.44***  (1.41 – 1.48) | 1.44***  (1.41 – 1.48) |
| Class: Non-manual | 1.27***  (1.24 – 1.29) | 1.21***  (1.19 – 1.24) | 1.21***  (1.19 – 1.24) |
| Job Strain index |  | 6.73***  (5.95 – 7.61) | 6.70***  (5.92 – 7.58) |
| JSI*cancer |  |  | 3.23  (.62 – 16.88) |
| ^a^Outcome variable: 0= Not having risk of low employment (work income >1G), 1= Having risk of low employment (work income ≤1G)  ^b^Cancer: 0 = non-cancer, 1 = cancer  ^c^Education: Secondary and lower is reference group.  ^d^Occupational class: Upper non-manual is reference group.  ^*^ p: probability value (*** p< 0.001, ** p<0.01, * p<0.05); 95%CI: 95% confidence interval | | | |

| Table 10: Results for Cox regression analysis with work (>1.0 basic amounts in income from work) as outcome variable^a^, models included predictor variables cancer^b^, education level^c^, occupational class^d^, job strain index (JSI) and interaction term between JSI and cancer. Results reported for women | | | |
| --- | --- | --- | --- |
| Variables | Model 1 | Model 2 | Model 3 |
|  | Hazard ratio  (95%CI) | Hazard ratio  (95%CI) | Hazard ratio  (95%CI) |
| Cancer | 1.62***  (1.51 – 1.73) | 1.62***  (1.52 – 1.74) | 3.56***  (2.31 – 5.51) |
| Education: High school | .64***  (.63 - .65) | .64***  (.63 - .65) | .64***  (.63 - .65) |
| Education: University and colleges (4 years) | .38***  (.38 - .39) | .38***  (.37 - .38) | .38***  (.37 - .38) |
| Education: University and colleges (>4 years) | .26***  (.25 - .27) | .27***  (.26 - .28) | .27***  (.26 - .28) |
| Class: Manual | 1.84***  (1.81 – 1.88) | 1.76***  (1.73 – 1.79) | 1.76***  (1.73 – 1.79) |
| Class: Non-manual | 1.28***  (1.27 – 1.3) | 1.25***  (1.24 – 1.27) | 1.25***  (1.23 – 1.27) |
| Job Strain Index |  | 2.34***  (2.09 – 2.62) | 2.38***  (2.13 – 2.67) |
| JSI*cancer |  |  | .10***  (.03- .35) |
| ^a^Outcome variable: 0= Not having risk of low employment (work income >1G), 1= Having risk of low employment (work income ≤1G)  ^b^Cancer: 0 = non-cancer, 1 = cancer  ^c^Education: Secondary and lower is reference group.  ^d^Occupational class: Upper non-manual is reference group.  ^*^ p: probability value (*** p< 0.001, ** p<0.01, * p<0.05); 95%CI: 95% confidence interval | | | |

| Table 11: Results for Cox regression analysis with work (>0.5 basic amounts in income from work) as outcome variable^a^, models included predictor variables cancer^b^, education level^c^, occupational class^d^, mechanical job exposure (MJE) and interaction term between MJE and cancer. Results reported for men | | | |
| --- | --- | --- | --- |
| Variables | Model 1 | Model 2 | Model 3 |
|  | Hazard ratio  (95%CI) | Hazard ratio  (95%CI) | Hazard ratio  (95%CI) |
| Cancer | 1.39***  (1.25 – 1.56) | 1.41***  (1.26 – 1.58) | 1.04  (.83 – 1.31) |
| Education: High school | .55***  (.54 - .56) | .55***  (.54 - .55) | .55***  (.54 - .55) |
| Education: University and colleges (4 years) | .41***  (.40 - .42) | .44***  (.43 - .45) | .44***  (.43 - .45) |
| Education: University and colleges (>4 years) | .26***  (.25 - .27) | .28***  (.27 - .30) | .28***  (.27 - .30) |
| Class: Manual | 1.73***  (1.69 – 1.78) | 1.55***  (1.51 – 1.59) | 1.55***  (1.51 – 1.59) |
| Class: Non-manual | 1.29***  (1.26 – 1.31) | 1.12***  (1.09 – 1.14) | 1.12***  (1.09 – 1.14) |
| Mechanical job exposure index |  | 2.13***  (2.02 – 2.25) | 2.12***  (2.01 – 2.24) |
| MJE*cancer |  |  | 3.30***  (1.59 – 6.85) |
| ^a^Outcome variable: 0= Not having risk of very low employment (work income > 0.5G), 1= Having risk of very low employment (work income ≤0.5G)  ^b^Cancer: 0 = non-cancer, 1 = cancer  ^c^Education: Secondary and lower is reference group.  ^d^Occupational class: Upper non-manual is reference group.  ^*^ p: probability value (*** p< 0.001, ** p<0.01, * p<0.05); 95%CI: 95% confidence interval | | | |

| Table 12: Results for Cox regression analysis with work (>0.5 basic amounts in income from work) as outcome variable^a^, models included predictor variables cancer^b^, education level^c^, occupational class^d^, mechanical job exposure (MJE) and interaction term between MJE and cancer. Results reported for women | | | |
| --- | --- | --- | --- |
| Variables | Model 1 | Model 2 | Model 3 |
|  | Hazard ratio  (95%CI) | Hazard ratio  (95%CI) | Hazard ratio  (95%CI) |
| Cancer | 1.63***  (1.51 – 1.75) | 1.64***  (1.52 – 1.76) | 1.59***  (1.37 – 1.85) |
| Education: High school | .63***  (.62 - .64) | .63***  (.62 - .64) | .63***  (.62 - .64) |
| Education: University and colleges (4 years) | .38***  (.37 - .38) | .38***  (.37 - .38) | .38***  (.37 - .38) |
| Education: University and colleges (>4 years) | .25***  (.24 - .26) | .27***  (.25 - .28) | .27***  (.25 - .28) |
| Class: Manual | 1.86***  (1.83 – 1.89) | 1.61***  (1.57 – 1.64) | 1.61***  (1.57 – 1.64) |
| Class: Non-manual | 1.28***  (1.26 – 1.30) | 1.14***  (1.12 – 1.16) | 1.14***  (1.12 – 1.16) |
| Mechanical job exposure index |  | 2.04***  (1.91 – 2.17) | 2.03***  (1.91 – 2.17) |
| MJE*cancer |  |  | 1.12  (.62 – 2.06) |
| ^a^Outcome variable: 0= Not having risk of very low employment (work income > 0.5G), 1= Having risk of very low employment (work income ≤0.5G)  ^b^Cancer: 0 = non-cancer, 1 = cancer  ^c^Education: Secondary and lower is reference group.  ^d^Occupational class: Upper non-manual is reference group.  ^*^ p: probability value (*** p< 0.001, ** p<0.01, * p<0.05); 95%CI: 95% confidence interval | | | |

| Table 13: Results for Cox regression analysis with work (>0.5 basic amounts in income from work) as outcome variable^a^, models included predictor variables cancer^b^, education level^c^, occupational class^d^, job strain index (JSI) and interaction term between JSI and cancer. Results reported for men | | | |
| --- | --- | --- | --- |
| Variables | Model 1 | Model 2 | Model 3 |
|  | Hazard ratio  (95%CI) | Hazard ratio  (95%CI) | Hazard ratio  (95%CI) |
| Cancer | 1.39***  (1.25 – 1.56) | 1.40***  (1.25 – 1.56) | .94  (.53 – 1.65) |
| Education: High school | .55***  (.54 - .56) | .55***  (.55 - .56) | .55***  (.55 - .56) |
| Education: University and colleges (4 years) | .41***  (.40 - .42) | .40***  (.39 - .41) | .40***  (.39 - .41) |
| Education: University and colleges (>4 years) | .26***  (.25 - .27) | .26***  (.25 - .27) | .26***  (.25 - .27) |
| Class: Manual | 1.73***  (1.69 – 1.78) | 1.46***  (1.42 – 1.50) | 1.46***  (1.42 – 1.50) |
| Class: Non-manual | 1.29***  (1.26 – 1.31) | 1.23***  (1.20 – 1.26) | 1.23***  (1.20 – 1.25) |
| Job Strain Index |  | 7.17***  (6.28 – 9.19) | 7.13***  (6.24 – 8.14) |
| JSI*cancer |  |  | 3.55  (.61 – 20.62) |
| ^a^Outcome variable: 0= Not having risk of very low employment (work income > 0.5G), 1= Having risk of very low employment (work income ≤0.5G)  ^b^Cancer: 0 = non-cancer, 1 = cancer  ^c^Education: Secondary and lower is reference group.  ^d^Occupational class: Upper non-manual is reference group.  ^*^ p: probability value (*** p< 0.001, ** p<0.01, * p<0.05); 95%CI: 95% confidence interval | | | |

| Table 14: Results for Cox regression analysis with work (>0.5 basic amounts in income from work) as outcome variable^a^, models included predictor variables cancer^b^, education level^c^, occupational class^d^, job strain index (JSI) and interaction term between JSI and cancer. Results reported for women | | | |
| --- | --- | --- | --- |
| Variables | Model 1 | Model 2 | Model 3 |
|  | Hazard ratio  (95%CI) | Hazard ratio  (95%CI) | Hazard ratio  (95%CI) |
| Cancer | 1.63***  (1.51 – 1.75) | 1.63***  (1.52 – 1.76) | 3.87***  (2.41 – 6.20) |
| Education: High school | .63***  (.62 - .64) | .63***  (.62 - .64) | .63***  (.62 - .64) |
| Education: University and colleges (4 years) | .38***  (.37 - .38) | .37***  (.36 - .38) | .37***  (.36 - .38) |
| Education: University and colleges (>4 years) | .25***  (.24 - .26) | .26***  (.24 - .27) | .26***  (.24 - .27) |
| Class: Manual | 1.86***  (1.83 – 1.89) | 1.76***  (1.73 – 1.80) | 1.76***  (1.73 – 1.80) |
| Class: Non-manual | 1.28***  (1.26 – 1.30) | 1.24***  (1.22 – 1.26) | 1.24***  (1.22 – 1.26) |
| Job Strain Index |  | 2.65***  (2.35 – 3.00) | 2.70***  (2.39 – 3.05) |
| JSI*cancer |  |  | .09***  (.02 - .31) |
| ^a^Outcome variable: 0= Not having risk of very low employment (work income > 0.5G), 1= Having risk of very low employment (work income ≤0.5G)  ^b^Cancer: 0 = non-cancer, 1 = cancer  ^c^Education: Secondary and lower is reference group.  ^d^Occupational class: Upper non-manual is reference group.  ^*^ p: probability value (*** p< 0.001, ** p<0.01, * p<0.05); 95%CI: 95% confidence interval | | | |
